# Determinants Related to Oxidative Stress Parameters in Pediatric Patients with Type 1 Diabetes Mellitus

**DOI:** 10.3390/nu15092084

**Published:** 2023-04-26

**Authors:** Monika Grabia, Katarzyna Socha, Jolanta Soroczyńska, Artur Bossowski, Renata Markiewicz-Żukowska

**Affiliations:** 1 Department of Bromatology, Faculty of Pharmacy with the Division of Laboratory Medicine, Medical University of Białystok, Mickiewicza 2D Street, 15-222 Białystok, Poland; 2 Clinic of Pediatrics, Endocrinology, Diabetology with the Subdivision of Cardiology, Children’s University Clinical Hospital in Białystok, Waszyngtona 17 Street, 15-274 Białystok, Poland

**Keywords:** oxidative stress, diabetes mellitus type 1, adolescents, minerals, toxic elements, antioxidant intake, metabolic management, continuous glucose monitoring systems

## Abstract

Adequate glycemic management is one of the main goals in treating type 1 diabetes mellitus (T1DM) and preventing the early onset of diabetic complications. Improperly controlled diabetes mellitus (DM) will result in oxidative stress (OS) and lead to further related health issues. Therefore, the aim of this study was to evaluate the body’s ability to defend against OS depending on the duration of T1DM, metabolic management, antioxidant intake and modern glycemic monitoring systems (GMS). The study included 103 adolescents with T1DM aged 10–17 years. The control group consisted of 65 healthy peers. The patients’ blood was assayed for antioxidant enzymes, minerals and toxic elements. In addition, their dietary intake of antioxidant components was assessed. The T1DM group had higher total oxidant status, oxidative stress index and Cu/Zn ratio values, higher concentrations of malondialdehyde and lower total antioxidant status (TAS) and chromium, zinc, superoxide dismutase and catalase levels than their healthy peers. The comparison between GMS types revealed favorable changes in OS parameters for the flash and continuous systems. Furthermore, an effect of vitamin A and C dietary intake on serum TAS concentrations was detected. More than 82% of the patients with high TAS fulfilled the estimated average requirement norm for vitamin A, and more than 60% fulfilled the vitamin C requirement. In youths with T1DM, it is advisable to observe the antioxidant activity of the body to prevent the accelerated development of diabetic complications.

## 1. Introduction

Type 1 diabetes mellitus (T1DM) is an autoimmune disease that involves insufficient insulin secretion caused by the destruction of pancreatic islet β cells [1]. The primary method of treatment is intensive insulin therapy, adjusted to the individual patient according to their lifestyle and nutrition. Multiple daily injections (MDI) and continuous subcutaneous insulin infusion (CSII) are typically administered [2,3]. Flash glucose monitoring (FGM) or continuous glucose monitoring (CGM) systems are becoming widely available modern approaches to monitoring blood glucose levels. In addition to improving the quality of life, they also reduce the number of hypo- and hyperglycemic incidents, which has a positive effect on the body [1,3,4]. Proper glycemic control is one of the key elements of preventing the progression of diabetic complications, which affects the antioxidant defense (AOD) system [5]. The body has developed a protective system against reactive oxygen species (ROS) in the form of enzymatic and non-enzymatic systems to guard against the deleterious effects of oxygen metabolism. The former relies on the cooperation of enzymes (including superoxide dismutase (SOD), catalase (CAT) and glutathione peroxidase (GPx)) to neutralize ROS, preventing damage to vital cellular structures. Additional, but certainly key, compounds that significantly affect this antioxidant potential of the body include dietary antioxidant nutrients (vitamins E and C, β-carotene, copper (Cu), zinc (Zn) and selenium (Se)) [6,7]. Other factors that influence the burden on the AOD system and cause oxidative damage to the above-mentioned enzymes and lipids (enhanced malondialdehyde (MDA) production) include exposure to arsenic (As), cadmium (Cd), mercury (Hg) and lead (Pb) [8,9]. Although the interest in oxidative stress in diabetes has been growing in recent years [5,10,11,12], there is a lack of research in the scientific world regarding a comprehensive approach in adolescents with T1DM that uses the dietary intake of nutrients with antioxidant potential and considers other possible contributors of oxidative stress (OS).

These mechanisms contributed to the aim of this study, which is to evaluate the body’s antioxidant capabilities in relation to T1DM duration, metabolic management, antioxidant intake and modern glucose monitoring systems.

## 2. Materials and Methods

### 2.1. Study Group

This case–control study involved 168 patients with T1DM and healthy peers aged 10–17 years. The participants in the T1DM group (*n* = 103) were under the care of the Department of Paediatrics, Endocrinology and Diabetology with the Subdivision of Cardiology of the Children’s University Clinical Hospital in Białystok, Poland, between March 2020 and September 2022. The diagnosis of T1DM was performed by doctors in the field of diabetology under the standards of the frequent presence of autoantibodies [1]. The inclusion criteria were an age of 10 to 17 years, a diagnosis of T1DM and the willingness to take part in the study. We excluded patients who had other types of diabetes mellitus (DM) or severe chronic diseases (e.g., cancer, hepatic disease and cardiac disease). The control group consisted of 65 individuals who volunteered at the Department of Bromatology of the Medical University of Białystok and declared no history of symptoms that would indicate the presence of DM or other chronic diseases at the time of recruitment. The Bioethics Committee’s approval (No. R-I-002/587/2019) was obtained to carry out the research. The consent of the participants’ guardian was required for participation.

### 2.2. Blood Sample Analysis of Selected Biomarkers

Vacutainer tubes containing a clot activator and gel or anticoagulant K2EDTA (Becton Dickinson, France) were used to collect fasting blood samples. After centrifugation (Centrifuge M-diagnostic, MPW, Warsaw, Poland) of the blood (10 min at about 2000 rpm), the serum was transferred into tubes and stored at −20 °C (to determine the elements) and at −80 °C (to measure the antioxidant defense and oxidative stress parameters). To prepare samples for the determination of Cu, chromium (Cr), Zn, As, Cd and Pb, the material was deproteinized (1 mol/L nitric acid, Suprapur, Merck, Darmstadt, Germany) and a surfactant was used (1% Triton X-100, Sigma-Aldrich, St Louis, MO, USA). The material was centrifuged for 10 min at about 6000 rpm (Centrifuge IKA mini G, IKA, Staufen im Breisgau, Germany). Zn, Cr, As and Pb were measured in the supernatant, while Cu and Cd were measured in a sample further diluted with 0.1 mol/L nitric acid. Shortly before the Se assay, the serum was diluted with 0.2% Triton X-100. Calibration curves were made using stock solutions (Merck, Darmstadt, Germany).

The concentrations of mineral components were measured using atomic absorption spectrometry (AAS) with Zeeman background correction (Z-2000, Hitachi, Japan). An acetylene/air flame atomization technique was used to measure Zn, and electrothermal atomization in a graphite cuvette was used for Cu, Se, Cr, Cd and Pb. Inductively coupled plasma mass spectrometry (ICP-MS, NexION300D, PerkinElmer, Waltham, MA, USA) with a kinetic energy discrimination chamber (KED) was used to determine As in whole blood. Hg was assayed directly in the material through AAS using the amalgamation technique (AMA-254, Leco Corp., Altec Ltd., Prague, Czechia). Moreover, a molar ratio was computed between Cu and Zn. The detection limits of the methods were 0.011 µg/L (As), 0.08 µg/L (Cd), 0.0005 mg/L (Cu), 0.12 µg/L (Cr), 0.003 µg/L (Hg), 0.97 µg/L (Pb), 1.44 µg/L (Se) and 0.02 mg/L (Zn). A spectrophotometry technique via a microplate reader (Infinite M200 Pro Tecan, Männedorf, Switzerland) was used to assay oxidative stress parameters such as total oxidant status (TOS; according to the methodology described by Erel et al. [13]), total antioxidant status (TAS) and GPx (with reagent kits from Randox Laboratories, Crumlin, County Antrim, UK) and MDA, CAT and SOD (with reagent kits from Cayman Chemical Company, Ann Arbor, MI, USA). Table 1 provides details of the determination. The oxidative stress index (OSI) is expressed as the ratio of TAS to TOS. Categories were defined for each level of TAS, TOS, OSI and Cu/Zn parameters to present the results in a transparent form. To classify the outcomes in the ‘low’ and ‘high’ categories, a reference range for a given parameter was defined and named as ‘medium’, which covered the tolerable normal values including the margin of error. The ranges were 1.2–1.45 mmol/L for TAS, 5–8 μmol H_2_O_2_ Equiv./L for TOS, 0.3–0.6 for OSI and 0.6–1.0 for Cu/Zn ratio. Glycated hemoglobin (HbA1c) was determined through ion exchange high-performance liquid chromatography using a Bio-Rad D-10TM system (Bio-Rad, Hercules, CA, USA). Certified materials specified for each kit were used to control the accuracy of the determination methods used (Seronorm Trace Elements Whole Blood L-2; Seronorm Trace Elements Serum L-1, Sero AS, Norway; Quality Control Randox; Catalase Control CaymanChem).

### 2.3. Nutritional Status and Nutrient Intake

Basic anthropometric measurements (body height and weight) were taken in order to assess the nutritional status. A questionnaire covering general information, medical history, insulin therapy and dietary habits was conducted with each patient. To estimate the precise dietary intake of nutrients with antioxidant potential, a nutritional interview was additionally performed, the detailed process of which was described in a previous publication [14].

### 2.4. Statistical Analysis

Statistical analysis of the data was carried out in the software program Statistica (version 13 PL; TIBCO Software Inc., Palo Alto, CA, USA). In order to determine the normality of the variables’ distribution so as to appropriately present the data and select adequate statistical tests, the Shapiro–Wilk, Kolmogorov–Smirnov and Lilliefors tests were performed. For quantitative variables, the Mann–Whitney U test and Kruskal–Wallis ANOVA test with post hoc analysis were applied. Correlations between parameters were checked with Spearman’s correlation coefficient. Multiple correspondence analysis (MCA) was used to detect all common features among individuals according to their antioxidant status and insulin therapy, along with the use of modern GMS and the duration of T1DM. The number of dimensions reliably representing the data was determined using a scree plot; the data were then analyzed and presented as a Burt matrix. Receiver operating characteristic (ROC) curve analysis was used to assess the diagnostic utility of antioxidant defense and oxidative stress markers. The overall efficacy of the indicator was expressed by the area under the ROC curve (AUC) with 95% confidence intervals (CI) and *p*-values. Cut-off values, sensitivity and specificity, the Youden index and positive and negative likelihood ratios were calculated. *p*-values of < 0.05 were recognized as statistically significant.

## 3. Results

Table 2 provides information on the research participants. For those with T1DM, details of the treatment are additionally included. Table 3 shows a comparison of the median concentrations of parameters in the blood between adolescents with T1DM and healthy peers. Among diabetics, statistically significantly higher values were found for Cu/Zn (*p* < 0.05), MDA (*p* < 0.01), TOS and OSI (*p* < 0.001), while lower values were found for Cr, Zn, TAS and SOD (*p* < 0.001), and CAT (*p* < 0.01). Only for MDA were there statistically significant differences between boys and girls in the T1DM group (3.863 µmol/L vs. 4.159 µmol/L, *p* < 0.05). Concerning the comparison between genders in the control group, discrepancies were found for Cd (0.728 µg/L vs. 1.173 µg/L, *p* < 0.05, respectively) and GPx (2160 U/L vs. 1206 U/L, *p* < 0.05, respectively). For the other parameters in Table 3 and for the dietary intake of nutrients with antioxidant potential, no such changes were observed.

Comparing those with newly diagnosed T1DM and those with the disease for more than two years (Table S1), the former group showed lower median Cd (0.629 µg/L vs. 0.784 µg/L, *p* < 0.01) and MDA (3.341 µmol/L vs. 4.171 µmol/L, *p* < 0.05) values, and higher Hg (0.680 µg/L vs. 0.391 µg/L, *p* < 0.01) and SOD (1.678 U/mL vs. 1.396 U/mL, *p* < 0.01) values. No differences were found between insulin therapies, while some were observed between individuals using modern GMS compared to traditional glucometers (Table S2). The participants using a CGM rather than a glucometer demonstrated higher TAS values (1.419 mmol/L vs. 1.236 mmol/L, respectively, *p* < 0.05) and lower OSI values (0.552 vs. 0.706, respectively, *p* < 0.01). Table S3 presents the concentrations of the markers in relation to the range in HbA1c values. Higher TAS levels were recorded in diabetics with an HbA1c level below 7% than in those with a level ranging from 7% to 9.9% (1.432 mmol/L vs. 1.259 mmol/L, *p* < 0.01) or above 10% (1.432 mmol/L vs. 1.299 mmol/L, *p* < 0.01). Similarly, OSI was noted to be significantly higher in patients with poor metabolic management (<7% vs. 7–9.9%: 0.470 vs. 0.631, *p* < 0.001; <7% vs. ≥10%: 0.470 vs. 0.739, *p* < 0.001). Furthermore, statistically significantly higher TOS and lower Cu values were found in individuals with elevated HbA1c.

The participants with T1DM were classified as having low, medium and high serum OS marker concentrations. Table 4 shows the medians of HbA1c values by category. There was a statistically significant difference between the HbA1c of diabetics with low and high TAS (9.0% vs. 6.9%, *p* < 0.01), as well as between those with low and medium TOS (6.6% vs. 7.7%, p < 0.05) and low and high TOS (6.6% vs. 8.1%, *p* < 0.05). The vast majority were classified as having medium TAS (41%), TOS (47%) and OSI (41%) values. More than half (52%) had a high Cu/Zn ratio, and only 28% had a high TAS. However, just 11% had a low TOS score, while 27% had a low OSI and 13% a low Cu/Zn ratio.

Table 5 shows the dietary intakes and percentages of individuals fulfilling the antioxidant nutrient dietary norms. Diabetics, compared to healthy peers, showed a statistically significantly lower intake of vitamin A (722 µg vs. 882 µg, *p* < 0.01) and β-carotene (2574 µg vs. 3522 µg, *p* < 0.01). The highest percentages of participants with intake below the norm were observed for vitamin E (T1DM: 88%; control: 85%), vitamin C (42% and 38%, respectively), zinc and vitamin A (23% each in T1DM and 26% and 14% in the control group). A small percentage of individuals consumed zinc and vitamin A above the upper tolerable intake level. Furthermore, a relationship between dietary intake of vitamin A and C and serum TAS concentrations was noted. More than 82% of the patients with high TAS fulfilled the estimated average requirement (EAR) for vitamin A, and 62% reached the EAR for vitamin C. These percentages were substantially lower in the other (low and medium) TAS categories.

A correlation was observed between selected parameters in the blood and also the intake of nutrients with antioxidant properties among diabetics (Table 6). A statistically significant positive association was found between HbA1c and OSI, and there was a negative one with TAS. Moreover, there was also a statistically significant positive correlation between Se and the dietary intake of vitamin A and β-carotene, as well as serum levels of TAS and CAT. In contrast, a statistically significant correlation was observed between the Cu/Zn ratio and the dietary intake of Zn, Cu, Mn, retinol and vitamin E.

**Figure 1:**
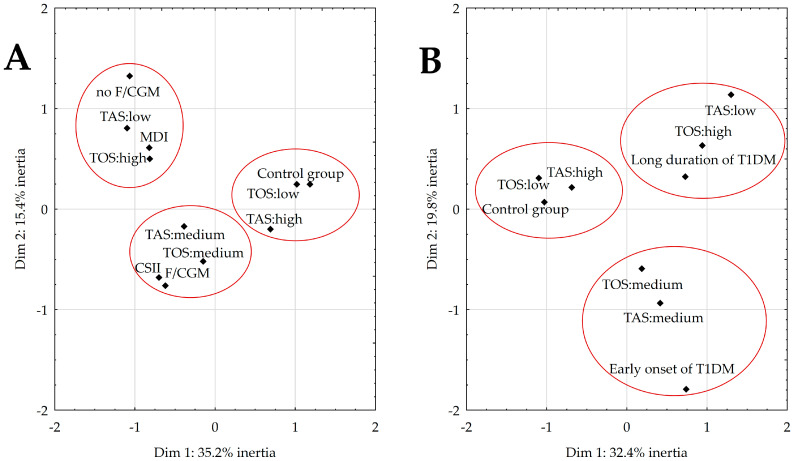
Multivariate correspondence analysis coordinate plot of total antioxidant and oxidant status, as well as insulin therapy with modern glycemic monitoring (**A**) and the duration of T1DM (**B**). Abbreviations: continuous glucose monitoring (CGM), continuous subcutaneous insulin infusion (CSII), flash glucose monitoring (FGM), multiple daily injections (MDI), type 1 diabetes mellitus (T1DM), total antioxidant status (TAS), total oxidant status (TOS).

The MCA helped describe the relation between the body’s antioxidant capabilities and insulin therapy with a modern GMS (Figure 1A) and the duration of T1DM (Figure 1B). Figure 1A,B depict the MCA results in bivariate form, which explains more than half of the total variation. For Figure 1A, the following three groups were identified:

(1) The first and fourth quadrants represent healthy peers with an above-average TAS range and low TOS.
(2) The second quadrant includes diabetics with low TAS and high TOS who were not supported by FGM or CGM or who used MDI.
(3) The final quadrant (III) consists of individuals with T1DM, who had medium TAS and TOS and who used FGM or CGM and CSII.

Figure 1B illustrates the following:

(1) In the first quadrant are individuals who have had T1DM for more than 2 years, with low TAS and high TOS.
(2) The second quadrant classifies healthy participants with an above-normal TAS and low TOS.
(3) The last quadrant (IV) contains recently diagnosed patients with medium TAS and TOS.

The ROC curve analysis revealed that TAS, TOS and OSI may be good predictors of oxidative stress for T1DM (Table 7). The calculated cut-off values corresponded to the highest accuracy of the markers, revealing a significant diagnostic potential for OSI (AUC: 0.87, *p* < 0.001) and TOS (AUC: 0.83, *p* < 0.001) and TAS (AUC: 0.77, *p* < 0.001). Comparative analysis between individuals with T1DM and the control group showed a significantly higher percentage of adolescents with elevated TOS (78% vs. 23%) and OSI (88% vs. 26%), as well as lower TAS (30% vs. 77%).

## 4. Discussion

Our study showed that adolescents with T1DM had lower levels of Zn (0.891 mg/L vs. 0.979 mg/L, *p* < 0.001) and Cu (0.874 mg/L vs. 0.903 mg/L, *p* > 0.05), but higher Cu/Zn ratios (1.057 vs. 0.981, *p* < 0.05) compared to their healthy peers. Similar results of Zn and higher Cu concentration in young diabetics were obtained by Salmonowicz et al. [11] (Zn: 0.88 mg/L; Cu: 1.26 mg/L) and Rychter-Stoś et al. [15] (0.946 mg/L and 1.333 mg/L, respectively). This finding is important for this population because of the functions that these elements are involved in. Zn is stored with insulin in the secretory vesicles of pancreatic islet β cells, and is responsible for the normal synthesis and secretion of insulin and glucagon [16]. Similarly, Cu is crucial for the normal activity of antioxidant enzymes such as SOD. Nevertheless, its homeostasis needs to be controlled, not only at the cellular level, but also in organs and tissues [17]. Although these values are within the reference range, a higher Cu/Zn ratio may indicate the possibility of oxidative stress in the body. Excessively high concentrations will lead to the formation of ROS and associated damage.

The above-mentioned elements are the main ones included in the active center of SOD, one of the most effective enzymes for neutralizing superoxide anions. Along with CAT, both are crucial enzymes of the AOD system. There is a significant rise in CAT activity at the onset of T1DM and a decline as it progresses. The opposite trend was observed for metabolic management: with higher HbA1c values, CAT levels increased [10]. Much the same was observed in our study. In addition, CAT positively correlated with Se, TAS and SOD levels. The concentrations of SOD were statistically significantly lower in diabetics than in healthy peers, and declined with the duration of T1DM. This lower activity level may also have been caused by diminished levels of Zn and Cu, which are embedded in the active center of this enzyme. More importantly, Zn is responsible for insulin synthesis in cells that are degraded in T1DM [16].

Selenium has a strong antioxidant potential that affects the antioxidant balance in the body [18]. In the current study, we found no differences in Se concentrations between adolescents with T1DM and healthy peers, similarly to Salmonowicz et al. [11]. However, they were within the reference range. One of the most important selenoenzymes is GPx. Its key function is to reduce hydrogen peroxide to water [18]. Darenskaya [19] reported a decrease in GPx activity among diabetics, which we also found in this study. Moreover, in our participants, it was about 20% lower in patients with long duration of T1DM as compared to the early onset group. This finding may be due to low glutathione levels or inactivation of the enzyme as a result of intense oxidative stress [20].

Even in low amounts, Pb may affect attention and concentration, and may cause irritability [9]. We reported lower levels of Pb in adolescents with T1DM than in the control group. Likewise, Forte et al. [21] observed that low concentrations of Zn, Pb, Cr, Mn and Ni were associated with T1DM. Similarly, in the case of the other elements we measured (Hg, Cd and As), no significant differences were found; the only exception was the statistically significant two-fold lower Cr values among diabetics. Low concentrations of Cr, as compared to a control group, were also observed by Lin et al. [22]. This is a disturbing sign, since Cr is involved in regulating glucose metabolism [23]. Furthermore, in a study on umbilical cord blood from babies, it was shown that the blood of patients who developed T1DM later in life had higher concentrations of Hg and As than a control group. One theory is that exposure to toxic metals during pregnancy may be one of many environmental factors that contributes to the future disease process [24].

Some of the by-products of lipid peroxidation are thiobarbituric acid reactive substances (TBARS). Using the thiobarbituric acid reagent, it is possible to measure MDA, a reactive aldehyde produced during the peroxidation of polyunsaturated fatty acids [25]. Diabetic patients were shown to be more susceptible to this production, further compounded by chronic hyperglycemia, which results in elevated production of free radicals and other ROS [12]. Similar findings were observed in our study. More importantly, MDA levels were higher in diabetics with an HbA1c level above 10% than in those with a level below 7%. A similar pattern was noted when individuals with T1DM for more than 2 years were compared to those with recently diagnosed T1DM.

The above-mentioned outcomes suggest the possibility of oxidative stress. For a comprehensive view, TAS and TOS were also determined, and their ratio, i.e., OSI, was calculated. Lower TAS and higher TOS and OSI indicate an oxidant–antioxidant imbalance through an endogenous antioxidant exhaustion, which leads to the manifestation of oxidative stress among young diabetics [14]. We observed this phenomenon, and it was particularly associated with deteriorated metabolic management measured via HbA1c, but also among individuals not using FGM or CGM. Moreover, considering their concentrations the highest oxidative stress was detected in pediatric participants at the initial stage of T1DM; this decreased amongst those with T1DM for more than 2 years.

It is also necessary to evaluate this study for its weaknesses and strengths. The type of research introduces a limitation, which is the inability to determine the exact causes of correlations and statistical significance; we can only identify them and make their presence known. The findings demonstrate that there is a strong need for research that can identify cause-and-effect relationships. It should be emphasized that a major strength of this study is the extensive comparison of the data regarding data classification, type of insulin therapy and use of modern FCM and CGM. Another advantage is the inclusion of a control group as part of the study, which made the comparative analysis possible and reliable.

## 5. Conclusions

Based on the findings of our research, it can be concluded that a low defense potential of the antioxidant system leading to high oxidative stress occurs among young diabetics and is present even at the onset of T1DM. In addition, there was a similar tendency in relation to deteriorating metabolic management, but not in patients supported by FGM or CGM. All these findings highlight the importance of monitoring the undesirable changes associated with high oxidant levels at T1DM onset, as they interfere with treatment and can lead to earlier progression of diabetic complications.

## Figures and Tables

**Table 1:** Detailed specifications for the elements, antioxidant defense markers and oxidative stress markers measured.

Parameter	Unit	Wavelength	Material
Cu	mg/L	324.8 nm	Serum
Cr	µg/L	359.3 nm	Serum
Se	µg/L	196 nm	Serum
Zn	mg/L	213.9 nm	Serum
TAS	mmol/L	600 nm	Serum
SOD	U/mL	450 nm	Serum
CAT	n/mol/min	540 nm	Serum
GPx	U/L whole blood	340 nm	Whole blood
TOS	μmol H_2_O_2_ equiv./L	560/800 nm	Serum
MDA	µmol/L	535 nm	Serum
As	µg/L	As^75^ 74.92 U *	Whole blood
Cd	µg/L	228.8 nm	Whole blood
Hg	µg/L	254 nm	Whole blood
Pb	µg/L	283.3 nm	Whole blood

* Isotope mass. Abbreviations: arsenic (As), catalase (CAT), cadmium (Cd), copper (Cu), chromium (Cr), glutathione peroxidase (GPx), mercury (Hg), malondialdehyde (MDA), lead (Pb), selenium (Se), superoxide dismutase (SOD), total antioxidant status (TAS), total oxidant status (TOS), zinc (Zn).

**Table 2:** Characteristics of the study cohort.

Parameter		T1DM Group (*n* = 103)	Control Group (*n* = 65)
Age (years)	Me (Q_1_–Q_3_)	13 (11–15)	15 (14–15)
Height (cm)		164 (155–173)	167 (158–178)
Body weight (kg)		57 (46–67)	56 (48–67)
HbA1c (%)		8 (6–10)	–
Age at diagnosis (years)		9 (6–11)	–
T1DM duration (years)		4 (1–7)	–
Gender (girls/boys)	%	51/49	38/62
Newly diagnosed (<2 years)		27	–
Type of insulin therapy (MDI/CSII)		41/59	–
Type of glucose monitoring system (Glucometer only/FGM/CGM)		29/41/30	–

Values are expressed as median and interquartile range (Me (Q_1_–Q_3_)) or percentage of subjects (%). Abbreviations: continuous glucose monitoring (CGM), continuous subcutaneous insulin infusion (CSII), flash glucose monitoring (FGM), glycated hemoglobin (HbA1c), multiple daily injections (MDI), type 1 diabetes mellitus (T1DM).

**Table 3:** Comparison of elements, antioxidant defense and oxidative stress markers between T1DM and control group.

Parameter	T1DM (*n* = 103)	Control (*n* = 65)	*p*-Value (T1DM vs. Control)
Cu (mg/L)	0.874 (0.724–1.154)	0.903 (0.706–1.130)	NS
Cu/Zn ratio	1.057 (0.842–1.458)	0.981 (0.669–1.179)	<0.05
Cr (µg/L)	0.648 (0.568–0.960)	1.530 (1.088–1.809)	<0.001
Se (µg/L)	60.9 (50.2–69.8)	61.4 (51.1–69.0)	NS
Zn (mg/L)	0.891 (0.796–1.020)	0.979 (0.898–1.119)	<0.001
TAS (mmol/L)	1.304 (1.173–1.525)	1.580 (1.476–1.761)	<0.001
SOD (U/mL)	1.470 (1.068–2.049)	2.114 (1.676–2.356)	<0.001
CAT (n/mol/min)	43.2 (27.7–72.4)	58.3 (48.2–75.2)	<0.01
GPx (U/L)	1329 (756–2168)	1749 (967–3049)	NS
TOS (μmol H_2_O_2_ Equiv./L)	7.568 (6.0–9.295)	4.847 (3.851–5.839)	<0.001
OSI	0.575 (0.439–0.771)	0.284 (0.241–0.382)	<0.001
MDA (µmol/L)	3.912 (2.512–5.312)	2.520 (1.690–4.950)	<0.01
As (µg/L)	0.593 (0.385–0.766)	0.581 (0.480–0.658)	NS
Cd (µg/L)	0.696 (0.577–1.330)	0.862 (0.569–1.650)	NS
Hg (µg/L)	0.456 (0.246–0.767)	0.344 (0.243–0.562)	NS
Pb (µg/L)	22.9 (15.2–32.6)	27.1 (20.1–31.5)	NS

Values are expressed as median and interquartile range (Me (Q_1_–Q_3_)). Statistically significant differences between the medians were detected using the Mann–Whitney U test. Abbreviations: arsenic (As), catalase (CAT), cadmium (Cd), chromium (Cr), copper (Cu), glutathione peroxidase (GPx), mercury (Hg), malondialdehyde (MDA), non-significant (NS), oxidative stress index (OSI), lead (Pb), selenium (Se), superoxide dismutase (SOD), type 1 diabetes mellitus (T1DM), total antioxidant status (TAS), total oxidant status (TOS), zinc (Zn).

**Table 4:** The level of HbA1c and the percentage of individuals with T1DM classified into each group according to the level (low, medium and high) of each AOD and OS parameter (TAS, TOS, OSI and Cu/Zn).

Parameter	Low		Medium		High		*p*-Value	
	%	Me (Q_1_–Q_3_)	%	Me (Q_1_–Q_3_)	%	Me (Q_1_–Q_3_)	Low vs. Medium	Low vs. High
TAS	31	9.0 (7.4–10.7)	41	7.8 (6.6–9.8)	28	6.9 (6.3–8.7)	NS	<0.01
TOS	11	6.6 (6.1–7.9)	47	7.7 (6.8–9.8)	43	8.1 (7.0–10.6)	<0.05	<0.05
OSI	27	7.5 (6.6–9.8)	41	7.8 (6.6–10.8)	32	8.2 (7.3–9.8)	NS	NS
Cu/Zn	13	7.5 (6.73–10.5)	35	7.6 (6.6–9.7)	52	8.1. (6.7–9.9)	NS	NS

Values are expressed as median and interquartile range (Me (Q_1_–Q_3_)) or percentage of subjects (%). Statistically significant differences between the medians were detected using Kruskal–Wallis ANOVA tests with post hoc analysis. Abbreviations: antioxidant defense (AOD), copper (Cu), glycated hemoglobin (HbA1c), non-significant (NS), oxidative stress (OS), oxidative stress index (OSI), type 1 diabetes mellitus (T1DM), total antioxidant status (TAS), total oxidant status (TOS), zinc (Zn).

**Table 5:** Dietary intake of antioxidants and percentages of participants whose diets were in compliance with nutritional standards.

Parameter	T1DM (*n* = 103)				Control (*n* = 65)			*p*-Value (T1DM vs. Control)
	Me (Q_1_–Q_3_)	Percentage of Subjects (%)			Me (Q_1_–Q_3_)	Percentage of Subjects (%)		
		Type of Norm	Norm	Below (Above *) Norm		Norm	Below (Above *) Norm	
Cu (mg)	0.97 (0.77–1.1)	EAR	87	13	1.0 (0.88–1.3)	97	3	NS
Mn (mg)	3.4 (2.3–4.4)	AI	97	3	3.1 (2.8–4.4)	98	2	NS
Zn (mg)	9.0 (7.6–11)	EAR	77	23	9.6 (8.2–12)	74	26	NS
		UL	97	2 *		92	8 *	
Vitamin A (µg)	722 (568–899)	EAR	77	23	882 (689–1184)	86	14	<0.01
		UL	88	12 *		85	15 *	
Retinol (µg)	260 (197–367)	n/d	–	–	280 (215–340)	–	–	NS
β-carotene (µg)	2574 (1754–3594)	n/d	–	–	3522 (2479–5178)	–	–	<0.01
Vitamin C (mg)	65 (37–108)	EAR	58	42	70 (48–110)	62	38	NS
Vitamin E (mg)	5.5 (4.3–6.8)	AI	12	88	6.1 (4.5–7.9)	15	85	NS

Values are expressed as median and interquartile range (Me (Q_1_–Q_3_)) or percentage of subjects (%). Statistically significant differences between the medians were detected using the Mann–Whitney U test. Abbreviations: adequate intake (AI), copper (Cu), estimated average requirement (EAR), manganese (Mn), no data (n/d), non-significant (NS), type 1 diabetes mellitus (T1DM), tolerable upper intake level (UL), zinc (Zn).

**Table 6:** Correlations between blood parameters and dietary intake of nutrients among patients with T1DM.

Parameter 1	Parameter 2	R	*p*-Value
HbA1c	TAS	−0.3	<0.01
	OSI	0.3	<0.001
Zn	Cu	0.2	<0.05
Cu	Hg	0.3	<0.01
Cu/Zn ratio	Hg	0.2	<0.01
	Dietary Zn	−0.3	<0.001
	Dietary Cu	−0.3	<0.001
	Dietary Mn	−0.3	<0.001
	Dietary retinol	−0.3	<0.01
	Dietary vitamin E	−0.2	<0.05
Se	TAS	0.4	<0.001
	CAT	0.2	<0.01
	Dietary vitamin A	0.2	<0.01
	Dietary β-carotene	0.3	<0.01
TAS	Cd	−0.3	<0.01
	CAT	0.2	<0.01
	SOD	0.2	<0.05
Cd	OSI	0.2	<0.05
	MDA	0.3	<0.01
	GPx	−0.3	<0.01
CAT	SOD	0.4	<0.001

Statistically significant correlations were detected using Spearman’s correlation coefficient. Repeated correlations between parameters were removed from the table. Abbreviations: catalase (CAT), cadmium (Cd), copper (Cu), glutathione peroxidase (GPx), glycated hemoglobin (HbA1c), mercury (Hg), malondialdehyde (MDA), manganese (Mn), oxidative stress index (OSI), selenium (Se), superoxide dismutase (SOD), type 1 diabetes mellitus (T1DM), total antioxidant status (TAS), zinc (Zn).

**Table 7:** Assessment of TAS, TOS and OSI as possible indicators of oxidative stress in T1DM.

Parameter	TAS	TOS	OSI
AUC (CI)	0.77 (0.7–0.84)	0.83 (0.77–0.90)	0.87 (0.82–0.93)
*p*-Value AUC	<0.001	<0.001	<0.001
Cut-off point	1.450	5.892	0.375
Sensitivity	72%	79%	89%
Specificity	77%	77%	74%
Youden index	0.488	0.556	0.632
+LR	0.231	0.231	0.262
−LR	0.282	0.214	0.107

Abbreviations: area under curve (AUC), confidence interval (CI), positive likelihood ratios (+LR), negative likelihood ratios (−LR), oxidative stress index (OSI), type 1 diabetes mellitus (T1DM), total antioxidant status (TAS), total oxidant status (TOS).

## Data Availability

The data presented in this study are available on request from the corresponding author.

## Supplementary Materials

The following supporting information can be downloaded at: https://www.mdpi.com/article/10.3390/nu15092084/s1, Table S1. Comparison of antioxidant defense and oxidative stress parameters regarding duration of T1DM; Table S2. Comparison of antioxidant defense and oxidative stress parameters regarding insulin therapy and glucose monitoring systems; Table S3. Comparison of antioxidant defense and oxidative stress parameters regarding classification of HbA1c levels among T1DM patients.
