# Supplementary material for: Determinants Related to Oxidative Stress Parameters in Pediatric Patients with Type 1 Diabetes Mellitus
Source: Nutrients. 2023 Apr 26;15(9):2084. doi: 10.3390/nu15092084 (PMC10180949; doi:10.3390/nu15092084)
Supplement: Supplementary file 1 [file nutrients-15-02084-s001.zip › nutrients-2323033-supplementary.pdf]

nutrients

Supplementary Material

Determinants Related to Oxidative Stress Parameters in Pediatric Patients with Type 1 Diabetes Mellitus

Monika Grabia [1,*], Katarzyna Socha [1], Jolanta Soroczyńska [1], Artur Bossowski [2] and Renata Markiewicz-Żukowska [1]

[1] Department of Bromatology, Faculty of Pharmacy with the Division of Laboratory Medicine, Medical University of Białystok, Mickiewicza 2D Street, 15-222 Białystok, Poland
[2] Clinic of Pediatrics, Endocrinology, Diabetology with the Subdivision of Cardiology, Children's University Clinical Hospital in Białystok, Waszyngtona 17 Street, 15-274 Białystok, Poland
* Correspondence: monika.grabia.diet@gmail.com; Tel.: +48-85-748-5469

Table S1. Comparison of antioxidant defense and oxidative stress parameters regarding duration of T1DM.

Parameter	Early onset of T1DM (n=28)	Long duration of T1DM (n=75)	*p*-Value
**Cu (mg/L)**	0.920 (0.731-1.532)	0.874 (0.724-1.079)	NS
**Cu/Zn ratio**	1.111 (0.884-1.632)	1.016 (0.821-1.420)	NS
**Cr (µg/L)**	0.634 (0.564-0.895)	0.652 (0.576-0.965)	NS
**Se (µg/L)**	62.5 (54.5-70.6)	60.3 (47.1-69.8)	NS
**Zn (mg/L)**	0.921 (0.803-1.059)	0.890 (0.796-1.008)	NS
**TAS (mmol/L)**	1.292 (1.157-1.400)	1.321 (1.173-1.567)	NS
**SOD (U/ml)**	1.678 (1.327-2.657)	1.396 (1.032-1.855)	<0.01
**CAT (n/mol/min)**	44.0 (28.3-80.0)	43.2 (27.8-64.8)	NS
**GPx (U/L)**	1601 (627-2514)	1285 (803-2072)	NS
**TOS (µmol H_2O_2 Equiv./L)**	8.033 (6.189-10.5)	7.500 (5.897-9.189)	NS
**OSI**	0.652 (0.416-0.862)	0.552 (0.450-0.713)	NS
**MDA (µmol/L)**	3.341 (1.712-4.549)	4.171 (2.771-5.688)	<0.05
**As (µg/L)**	0.593 (0.385-0.762)	0.593 (0.358-0.766)	NS
**Cd (µg/L)**	0.629 (0.445-0.729)	0.784 (0.601-1.558)	<0.01
**Hg (µg/L)**	0.680 (0.350-0.983)	0.391 (0.185-0.744)	<0.01
**Pb (µg/L)**	20.8 (15.0-30.8)	23.1 (15.2-34.4)	NS

Values are expressed as median and interquartile range (Me (Q_1–Q_3). Statistically significant differences between the medians were detected by the Mann–Whitney U test. Abbreviations: arsenic (As), catalase (CAT), cadmium (Cd), chromium (Cr), copper (Cu), glutathione peroxidase (GPx), mercury (Hg), malondialdehyde (MDA), non-significant (NS), oxidative stress index (OSI), lead (Pb), selenium (Se), superoxide dismutase (SOD), type 1 diabetes mellitus (T1DM), total antioxidant status (TAS), total oxidant status (TOS), zinc (Zn).

Table S2. Comparison of antioxidant defense and oxidative stress parameters regarding insulin therapy and glucose monitoring systems.

	Insulin therapy		Glucose monitoring systems				
**Parameter**	**MDI (n=42)**	**CSII (n=61)**	**Glucometer only (n=30)**	**FGM (n=42)**	**CGM (n=31)**	**F/CGM (n=73)**	***p*-Value**
**Cu (mg/L)**	0.816 (0.695-1.114)	0.906 (0.784-1.230)	0.831 (0.724-1.049)	0.862 (0.709-1.404)	0.905 (0.792-1.079)	0.898 (0.737-1.230)	NS
**Cu/Zn ratio**	0.941 (0.735-1.474)	1.161 (0.890-1.447)	0.984 (0.821-1.447)	1.072 (0.752-1.590)	1.141 (0.920-1.420)	1.141 (0.886-1.464)	NS
**Cr (µg/L)**	0.652 (0.576-0.960)	0.643 (0.568-0.946)	0.678 (0.624-0.946)	0.633 (0.514-1.120)	0.638 (0.564-0.803)	0.636 (0.564-0.960)	NS
**Se (µg/L)**	61.9 (54.3-70.8)	60.3 (46.0-69.3)	61.2 (47.1-70.8)	61.1 (56.0-70.8)	60.3 (45.6-69.0)	60.9 (50.5-69.8)	NS
**Zn (mg/L)**	0.912 (0.825-1.026)	0.890 (0.733-0.960)	0.923 (0.821-1.037)	0.901 (0.825-1.020)	0.854 (0.711-0.954)	0.890 (0.784-1.008)	NS
**TAS (mmol/L)**	1.302 (1.154-1.407)	1.321 (1.174-1.596)	1.236 (1.068-1.367)	1.304 (1.220-1.553)	1.419 (1.201-1.691)	1.336 (1.213-1.602)	$<0.05^{A}$ $<0.01^{B,C}$
**SOD (U/ml)**	1.608 (1.215-2.174)	1.363 (1.032-1.855)	1.514 (1.120-2.690)	1.460 (1.056-1.869)	1.413 (1.068-2.161)	1.450 (1.068-1.885)	NS
**CAT (n/mol/min)**	50.7 (29.2-73.7)	42.1 (26.6-64.8)	44.5 (30.7-84.8)	39.9 (27.5-63.4)	53.3 (23.3-73.7)	42.6 (26.6-66.8)	NS
**GPx (U/L)**	1438 (756-2491)	1285 (817-2071)	1163 (629-2119)	1401 (728-2256)	1420 (904-2305)	1420 (849-2256)	NS
**TOS (µmol H_2O_2 Equiv./L)**	7.847 (5.892-9.216)	7.500 (6.189-9.295)	4.035 (3.323-5.382)	7.160 (5.676-8.865)	7.945 (5.9-10.1)	7.500 (5.841-9.514)	NS
**OSI**	0.575 (0.450-0.787)	0.586 (0.431-0.745)	0.706 (0.491-0.904	0.520 (0.388-0.652)	0.552 (0.412-0.713)	0.533 (0.411-0.659)	$<0.01^{A,C}$
**MDA (µmol/L)**	4.206 (2.877-5.300)	3.632 (2.265-5.335)	4.035 (3.324-5.382)	3.839 (2.559-4.759)	4.220 (1.841-5.594)	3.862 (2.241-5.276)	NS
**As (µg/L)**	0.593 (0.356-0.766)	0.593 (0.385-0.741)	0.593 (0.415-0.736)	0.595 (0.296-0.808)	0.596 (0.385-0.820)	0.596 (0.356-0.808)	NS
**Cd (µg/L)**	0.671 (0.559-1.454)	0.722 (0.59-1.078)	0.751 (0.571-1.893)	0.656 (0.581-0.926)	0.777 (0.585-0.969)	0.696 (0.585-0.964)	NS
**Hg (µg/L)**	0.576 (0.317-0.767)	0.363 (0.218-0.751)	0.636 (0.218-1.002)	0.570 (0.317-0.964)	0.342 (0.177-0.536)	0.421 (0.248-0.701)	NS
**Pb (µg/L)**	25.7 (14.6-32.6)	21.8 (16.2-31.3)	22.3 (16.2-47.5)	24.0 (14.6-32.6)	21.8 (15.2-28.6)	23.0 (15.2-31.0)	NS

Values are expressed as median and interquartile range (Me (Q_1–Q_3). Statistically significant differences between the medians (A – glucometer vs. FGM; B – glucometer vs. CGM; C – glucometer vs. FGM&CGM) were detected by the Mann–Whitney U test and Kruskal–Wallis ANOVA test with post-hoc analysis. Abbreviations: arsenic (As), catalase (CAT), cadmium (Cd), continuous glucose monitoring (CGM), chromium (Cr), continuous subcutaneous insulin infusion (CSII), copper (Cu), flash glucose monitoring (FGM), glutathione peroxidase (GPx), mercury (Hg), malondialdehyde (MDA), multiple daily injections (MDI), non-significant (NS), oxidative stress index (OSI), lead (Pb), selenium (Se), superoxide dismutase (SOD), total antioxidant status (TAS), total oxidant status (TOS), zinc (Zn).

Table S3. Comparison of antioxidant defense and oxidative stress parameters regarding classification of HbA1c levels among T1DM patients.

Parameter	HbA1c ≤ 7% (n=34)	HbA1c 7.1-9.9% (n=44)	HbA1c ≥ 10% (n=25)	*p*-Value
**Cu (mg/L)**	0.876 (0.724-1.150)	0.918 (0.797-1.329)	0.779 (0.635-1.012)	<0.05 [B]
**Cu/Zn ratio**	0.971 (0.745-1.474)	1.134 (0.911-1.470)	1.014 (0.882-1.377)	NS
**Cr (µg/L)**	0.676 (0.596-1.120)	0.636 (0.538-1.043)	0.624 (0.592-0.803)	NS
**Se (µg/L)**	63.6 (50.5-69.7)	59.9 (50.1-70.6)	61.6 (50.4-70.8)	NS
**Zn (mg/L)**	0.908 (0.840-1.030)	0.891 (0.776-1.035)	0.848 (0.671-0.960)	NS
**TAS (mmol/L)**	1.432 (1.279-1.648)	1.259 (1.142-1.417)	1.299 (0.923-1.394)	<0.01 [A, C]
**SOD (U/ml)**	1.475 (1.154-1.885)	1.386 (0.947-1.769)	1.564 (1.159-2.356)	NS
**CAT (n/mol/min)**	37.4 (26.6-66.8)	48.2 (29.6-78.2)	45.8 (27.5-63.8)	NS
**GPx (U/L)**	1351 (908-2168)	1259 (645-2092)	1606 (675-2258)	NS
**TOS (µmol H_2O_2 Equiv./L)**	6.657 (5.270-9.027)	7.870 (6.890-9.635)	8.135 (6.243-9.351)	<0.01 [A]
**OSI**	0.470 (0.376-0.574)	0.631 (0.508-0.784)	0.739 (0.467-0.936)	<0.001 [A, C]
**MDA (µmol/L)**	4.132 (2.323-4.700)	3.590 (1.805-5.294)	4.524 (3.323-6.465)	NS
**As (µg/L)**	0.597 (0.385-0.817)	0.593 (0.341-0.779)	0.593 (0.385-0.661)	NS
**Cd (µg/L)**	0.637 (0.494-1.124)	0.772 (0.645-1.351)	0.601 (0.564-1.302)	NS
**Hg (µg/L)**	0.488 (0.177-0.751)	0.404 (0.248-0.756)	0.513 (0.332-0.964)	NS
**Pb (µg/L)**	20.6 (16.2-27.2)	22.8 (14.2-31.3)	25.8 (17-35.2)	NS

Values are expressed as median and interquartile range (Me (Q_1–Q_3). Statistically significant differences between the medians (A – HbA1c ≤ 7% vs. 7.1-9.9%; B – HbA1c 7.1-9.9% vs. ≥ 10%; C – HbA1c ≤ 7% vs. ≥ 10%) were detected by the Kruskal–Wallis ANOVA test with post-hoc analysis. Abbreviations: arsenic (As), catalase (CAT), cadmium (Cd), chromium (Cr), copper (Cu), glutathione peroxidase (GPx), glycated hemoglobin (HbA1c), mercury (Hg), malondialdehyde (MDA), non-significant (NS), oxidative stress index (OSI), lead (Pb), selenium (Se), superoxide dismutase (SOD), type 1 diabetes mellitus (T1DM), total antioxidant status (TAS), total oxidant status (TOS), zinc (Zn).
